# When to stop septic shock resuscitation: clues from a dynamic perfusion monitoring

**DOI:** 10.1186/s13613-014-0030-z

**Published:** 2014-10-11

**Authors:** Glenn Hernandez, Cecilia Luengo, Alejandro Bruhn, Eduardo Kattan, Gilberto Friedman, Gustavo A Ospina-Tascon, Andrea Fuentealba, Ricardo Castro, Tomas Regueira, Carlos Romero, Can Ince, Jan Bakker

**Affiliations:** 1 Departamento de Medicina Intensiva, Facultad de Medicina, Pontificia Universidad Católica de Chile, Marcoleta 367, Santiago 8320000, Chile; 2 Unidad de Pacientes Críticos, Hospital Clínico, Universidad de Chile, Santos Dumont 999, Santiago 8380456, Chile; 3 Department of Critical Care Medicine, Hospital de Clínicas de Porto Alegre, Rua Ramiro Barcelos, 2350, Porto Alegre-RS, 90035-903, Brazil; 4 Intensive Care Unit, Fundación Valle del Lili, Av. Simón Bolívar Cra 98 # 18-49, Cali, Valle del Cauca, Colombia; 5 Department of Intensive Care Adults, Erasmus MC University Medical Centre, Doctor Molewaterplein 50-60, Rotterdam, the Netherlands

**Keywords:** Septic shock, Perfusion, Resuscitation, Lactate, Microcirculation

## Abstract

**Background:**

The decision of when to stop septic shock resuscitation is a critical but yet a relatively unexplored aspect of care. This is especially relevant since the risks of over-resuscitation with fluid overload or inotropes have been highlighted in recent years. A recent guideline has proposed normalization of central venous oxygen saturation and/or lactate as therapeutic end-points, assuming that these variables are equivalent or interchangeable. However, since the physiological determinants of both are totally different, it is legitimate to challenge the rationale of this proposal. We designed this study to gain more insights into the most appropriate resuscitation goal from a dynamic point of view. Our objective was to compare the normalization rates of these and other potential perfusion-related targets in a cohort of septic shock survivors.

**Methods:**

We designed a prospective, observational clinical study. One hundred and four septic shock patients with hyperlactatemia were included and followed until hospital discharge. The 84 hospital-survivors were kept for final analysis. A multimodal perfusion assessment was performed at baseline, 2, 6, and 24 h of ICU treatment.

**Results:**

Some variables such as central venous oxygen saturation, central venous-arterial pCO_2_ gradient, and capillary refill time were already normal in more than 70% of survivors at 6 h. Lactate presented a much slower normalization rate decreasing significantly at 6 h compared to that of baseline (4.0 [3.0 to 4.9] vs. 2.7 [2.2 to 3.9] mmol/L; *p* < 0.01) but with only 52% of patients achieving normality at 24 h. Sublingual microcirculatory variables exhibited the slowest recovery rate with persistent derangements still present in almost 80% of patients at 24 h.

**Conclusions:**

Perfusion-related variables exhibit very different normalization rates in septic shock survivors, most of them exhibiting a biphasic response with an initial rapid improvement, followed by a much slower trend thereafter. This fact should be taken into account to determine the most appropriate criteria to stop resuscitation opportunely and avoid the risk of over-resuscitation.

## 1 Background

Several clinical studies have demonstrated that persistent impairment of perfusion-related physiological variables is associated with increased mortality in septic shock patients [1]–[3]. Therefore, current guidelines recommend normalization of relevant physiologic variables such as lactate and/or central venous oxygen saturation (ScvO_2_) as resuscitation goals, basically through oxygen transport (DO_2_) optimization [4],[5]. In addition, peripheral perfusion, central venous-arterial pCO_2_ gradient (P(cv-a)CO_2_), and microcirculatory abnormalities have also been linked to morbidity or mortality and suggested as potential complementary targets [6]–[8].

However, the issue of when to stop resuscitation has become more relevant in recent years as the risks of over-resuscitation have also been increasingly highlighted. In fact, pursuing complete normalization of all potential perfusion-related goals with repeated attempts to increase DO_2_ could eventually result in severe adverse effects such as fluid overload, pulmonary edema, intra-abdominal hypertension, cardiac arrhythmias, and myocardial ischemia, thus possibly increasing morbidity and mortality [9]–[11].

From a physiological point of view, the problem is far more complex. For instance, it is not known if all perfusion-related variables are equally sensitive to DO_2_ optimization [12], a factor that could critically influence their specific normalization rates. Besides, parameters traditionally considered as reflecting tissue perfusion like lactate are also mechanistically determined by non-flow dependent or mixed mechanisms [13]. This may result in a wide variability on individual recovery time courses after optimization of DO_2_ depending on the predominant pathogenic mechanism. The practical aspect is that if a more likely flow-dependent parameter is selected as a goal (such as P(cv-a)CO_2_ or ScvO_2_), it may normalize earlier than a less flow-dependent one such as lactate. In other words, from a theoretical point of view, the resuscitation length could vary dramatically depending on these considerations leading eventually to the risk of over-resuscitation if the selected goal exhibits an intrinsic slow normalization rate.

To address this subject, we designed a prospective study to evaluate the specific normalization rates of several perfusion-related variables in a cohort of consecutive septic shock patients subjected to protocolized resuscitation and multimodal perfusion assessment. We *a priori* decided to include only ultimately hospital-surviving patients for analysis to provide a relevance perspective to persistent abnormalities after initial resuscitation.

## 2 Methods

### 2.1 Setting

We conducted a prospective observational study from July 2011 to November 2012 in a mixed 16-bed ICU at our university hospital. The institutional review board of our university approved this study and waived the need of an informed consent because of the observational nature of the study (Comité de Etica en Investigación, Facultad de Medicina, Pontificia Universidad Católica de Chile; approval number 11-113).

### 2.2 Patient selection

We included all consecutive adult patients admitted to the ICU with septic shock diagnosis according to the 2001 consensus definition [14], with a basal arterial lactate >2 mmol/L and full commitment for resuscitation.

### 2.3 Protocol and measurements

Patients were studied for the first 24 h following initiation of ICU-based resuscitation and were followed until death or hospital discharge. Clinical and demographic data and severity scores [15],[16] were collected for each patient at baseline (at inclusion = 0 h).

The following measurements as part of a multimodal perfusion assessment were obtained at baseline and at 2, 6, and 24 h after starting ICU resuscitation:

1. Macro-hemodynamic variables: mean arterial pressure (MAP), heart rate, norepinephrine (NE) or vasoactive drug doses, central venous pressure (CVP), pulse pressure variation (%), and pulmonary artery catheter-derived values (when in place). Fluid administration was also registered at each predefined time-point.

2. Metabolic-related perfusion variables: ScvO_2_, arterial lactate and P(cv-a)CO_2_.

3. Peripheral perfusion was assessed with the capillary refill time (CRT) (normal values ≤4.0 s) [17].

In a subgroup of patients who arrived within the first 2 h of onset of septic shock and were already in mechanical ventilation, we performed also the following microcirculatory and micro-oxygenation assessments:

1. Thenar muscle oxygen saturation (StO_2_) was measured by a tissue spectrometer (InSpectra Model 650, Hutchinson Technology, Minneapolis, MN, USA) [18]. A value ≥75% was considered as normal for this protocol. A vascular occlusion test (VOT) was performed as described elsewhere [19]. During the reperfusion phase of the VOT, the recovery slope of the StO_2_ signal was registered and calculated with a software (InSpectra V3-03, Hutchinson Technology, MN, USA) and expressed in percentage per second (values >3.5%/s were considered as normal for this study based on our own data in healthy volunteers (data not shown)).

2. Microcirculatory-derived variables: sublingual microcirculation was assessed with sidestream dark field video microscopy imaging (Microscan® for NTSC, MicroVision Medical, Amsterdam, the Netherlands). Image acquisition and analysis were performed following recent recommendations of a consensus conference [20]. A trained independent investigator performed image analysis in all cases, and these data were not disclosed to the attending physicians or considered for management. Parameters considered for this study were proportion of perfused vessels (PPV; values ≥90% were considered as normal); perfused vessel density (PVD; values ≥14 n/mm were considered as normal); and microcirculatory flow index (MFI; values ≥2.5 were considered as normal).

All patients were managed according to a local algorithm [21] aimed at macrohemodynamic stabilization and improvement of hypoperfusion abnormalities (both ScvO_2_ and lactate) during the first 24 h following implementation of adequate maneuvers for source control. The main strategy to improve oxygen transport/oxygen consumption unbalance was preload optimization. For this purpose, the algorithm included early fluid loading, followed by NE as needed to maintain a MAP >65 mmHg. Further fluid resuscitation was guided by dynamic predictors (pulse pressure variation) in patients under mechanical ventilation, except in patients with atrial fibrillation [22]. To assess pulse pressure variation in patients with acute respiratory distress syndrome, we transiently increased tidal volume to 8 mL/kg. A pulmonary artery catheter was placed in patients with high NE requirements (>0.3 mcg/kg/min) or past medical history of cardiac disease. In patients with atrial fibrillation and with a pulmonary artery catheter in place, volume administration was guided by a Starling curve approach in which progressive fluid boluses were administered until reaching a plateau in cardiac index. In patients with spontaneous breathing, fluid resuscitation was guided by central venous pressure criteria as suggested by current guidelines [4]. Dobutamine was restricted to patients with cardiac index <2.2 L/min/m^2^ in whom attending physicians had ruled-out hypovolemia as the cause of persistent hypoperfusion. Arterial hemoglobin oxygen saturation was maintained at >90%, and hemoglobin concentrations at 8 g/dl or higher to optimize arterial oxygen content. Mechanical ventilation settings were adjusted according to current recommendations [4]. Intra-abdominal pressure was monitored and treated according to recent recommendations [23].

### 2.4 Statistical analysis

Categorical data were analyzed with chi-square or Fisher's exact test when appropriate. Repeated measures were analyzed with Friedman test with Bonferroni post-hoc correction. All data are presented as medians and 25 to 75 interquartile ranges. We performed trend estimations of different perfusion and microcirculatory variables computing the average ranks for each variable using Pearson's correlation coefficient as described by Cuzick [24]. We also performed a normalization procedure for lactate, P(cv-a)CO_2_, and CRT values to rescaling them in order to allow comparison of these parameters' relative changes in specific time periods. For each variable, the highest normal value was taken, and each individual value was divided by it. Hence, medians and interquartile ranges were plotted. Fractional polynomials analyses were done to model realistic fitting curves for each parameter trend. All reported *p* values are two-sided, with a significant alpha level at 5%. SPSS 17 (SPSS Inc., Chicago, IL, USA) and Stata 12 (StataCorp LP, College Station, TX, USA) statistical packages were used for analyses.

## 3 Results

One hundred and four patients were admitted with a diagnosis of septic shock during the 18-month period, with a hospital mortality of 19% (*N* = 20).

Within this group, 84 were discharged alive from the hospital and constitute our definitive study group. Thus, data regarding non-survivors were not considered for final analysis and are only provided in Additional file 1: Table S1. Basal demographic, clinical, and physiological data and severity scores of the whole population and the 84 survivors are provided in Table 1. The main septic sources were abdominal (*n* = 45), pulmonary (*n* = 23), urinary tract (*n* = 8), and others, including soft tissue and catheter sources (*n* = 8). Sixteen patients were admitted directly from the operating room.

**Table 1:** General characteristics of the study population

**Parameters**	**Total patients**	**Survivors without MC**	**Survivors with MC**	***p*** **value between survivors**
Number	104	53	31	
Age (years)	66 [56 to 75]	63 [52 to 71]	67 [56 to 76]	0.4
Male/female (%)	45/55	47/53	39/61	0.3
APACHE II	23 [19 to 26]	23 [18 to 27]	24 [19 to 27]	0.5
Basal SOFA	10 [8 to 13]	10 [8 to 13]	10 [8 to 13]	0.9
24 h SOFA	10 [7 to 12]	10 [7 to 12]	11 [10 to 13]	0.6
Length of hospital stay (days)	23 [15 to 35]	24 [16 to 36]	24 [15 to 43]	0.8
Length of ICU stay (days)	11 [7 to 17]	11 [7 to 25]	10 [7 to 16]	0.5
MV duration (days)	8 [5 to 15]	9 [5 to 15]	8 [5 to 14]	0.4
Basal NE requirements (mcg/kg/min)	0.11 [0.03 to 0.30]	0.10 [0.04 to 0.28]	0.16 [0.03 to 0.34]	0.4
Basal lactate (mmol/L)	4.0 [3.0 to 4.9]	4.2 [3.0 to 5.3]	4.0 [2.8 to 4.9]	0.9

Values are expressed as median [interquartile range] or percentage. MC, microcirculatory assessment; APACHE II, acute physiology and chronic health evaluation II; SOFA, sequential organ failure assessment; ICU, intensive care unit; MV, mechanical ventilation; NE, norepinephrine.

Patients received 1,750 [640 to 2,400] mL of crystalloids in the pre-ICU setting after meeting septic shock criteria. The rate of fluid administration tended to decrease over time during ICU resuscitation. A total of 1,150 [600 to 1,600] mL of crystalloids were administered during the first 2 h, 980 [510 to 1,410] mL from 3 to 6 h, and 1,020 [290 to 2,400] mL from 7 to 24 h of ICU-based resuscitation. A pulmonary artery catheter was placed in 38 patients. Basal cardiac index and pulmonary arterial occlusion pressure were 3.1 [2.5 to 3.9] L/min/m^2^ and 17 [12 to 23] mmHg, respectively. Dobutamine was used in ten patients. Basal and 24-h intra-abdominal pressures were 13 [9 to 17] and 13 [8 to 15] mmHg, respectively.

By definition, all patients started ICU-based resuscitation with an abnormal lactate as compared to only 8, 31, 32, and 39 patients with abnormal ScvO_2_, StO_2_, P(cv-a)CO_2_, and CRT values, respectively. Medians values for individual macrohemodynamic and perfusion variables at different time-points in patients with abnormal values at baseline are shown in Table 2. Lactate decreased significantly from 0 to 6 h (4.0 [3.0 to 4.9] vs. 2.7 [2.2 to 3.9] mmol/L; *p* < 0.01).

**Table 2:** Evolution of different perfusion and hemodynamic parameters in a cohort of 84 hospital survivors

**Perfusion parameters**	**Number of patients with altered baseline values**	**Baseline**	**2 h**	**6 h**	**24 h**	***p*** **value**^**a**^
Lactate (mmol/L)	84	4.0 [3.0 to 4.9]	3.4 [2.4 to 4.2]	2.8 [2.0 to 3.8]	1.8 [1.4 to 2.5]	<0.001
P(cv-a)CO_2_ (mmHg)	34	8 [7 to 9]	6 [5 to 8]	5 [3 to 7]	4 [3 to 6]	<0.001
CRT (s)	43	6 [5 to 8]	4 [3 to 5]	3 [2 to 6]	2 [2 to 4]	0.001
ScvO_2_ (%)	8	62 [58 to 67]	65 [60 to 69]	71 [70 to 74]	74 [70 to 79]	0.001
**Hemodynamic parameters**	**Number of patients assessed**	**Baseline**	**2 h**	**6 h**	**24 h**	***p*** **value**^**a**^
CI (L/min/m^2^)	38	3.1 [2.5 to 3.9]	3.5 [2.9 to 4.6]	3.2 [2.6 to 3.8]	2.8 [2.4 to 4.1]	NS
Pulse pressure variation (%)	63	6 [3 to 8]	5 [2 to 8]	6 [2 to 8]	5 [4 to 9]	NS
CVP (mmHg)	84	13 [9 to 17]	14 [10 to 16]	14 [10 to 16]	14 [11 to 17]	NS
MAP (mm Hg)	84	73 [67 to 79]	71 [68 to 74]	71 [68 to 74]	72 [70 to 77]	NS
NE dose (mcg/kg/min)	84	0.11 [0.04 to 0.3]	0.18 [0.06 to 0.31]	0.17 [0.07 to 0.35]	0.05 [0 to 0.23]	NS
IAP (mmHg)	72	12 [9 to 15]	11 [9 to 13]	12 [9 to 12]	11 [8 to 14]	NS

Values are expressed as median [interquartile range]. ^a^Comparison within group of variables was made with non-parametric trend. p(cv-a)CO_2_, central venous to arterial pCO_2_ gradient; CRT, capillary refill time; ScvO_2_, central venous oxygen saturation; CI, cardiac index; CVP, central venous pressure; MAP, mean arterial pressure; NE, norepinephrine; IAP, intra-abdominal pressure.

**Figure 1:**
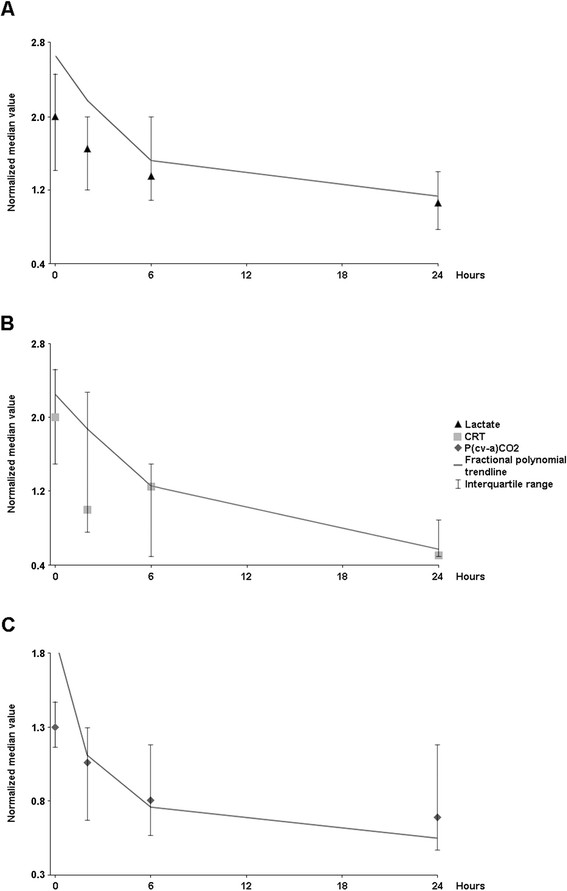
**Time-trend changes for selected perfusion parameters after normalization showing a biphasic recovery trend (see statistical analysis): A,** ***lactate;*** **B,** ***capillary refill time (CRT);*** **C,** ***central venous-arterial pCO***_***2***_ ***gradient (P(cv-a)CO***_***2***_**).**

When analyzing the time-trend changes of lactate, P(cv-a)CO_2_, and CRT values, a biphasic curve could be drawn (Figure 1) where a rapid decrease in every variable during the first 6 h was followed by a slower decay thereafter.

**Figure 2:**
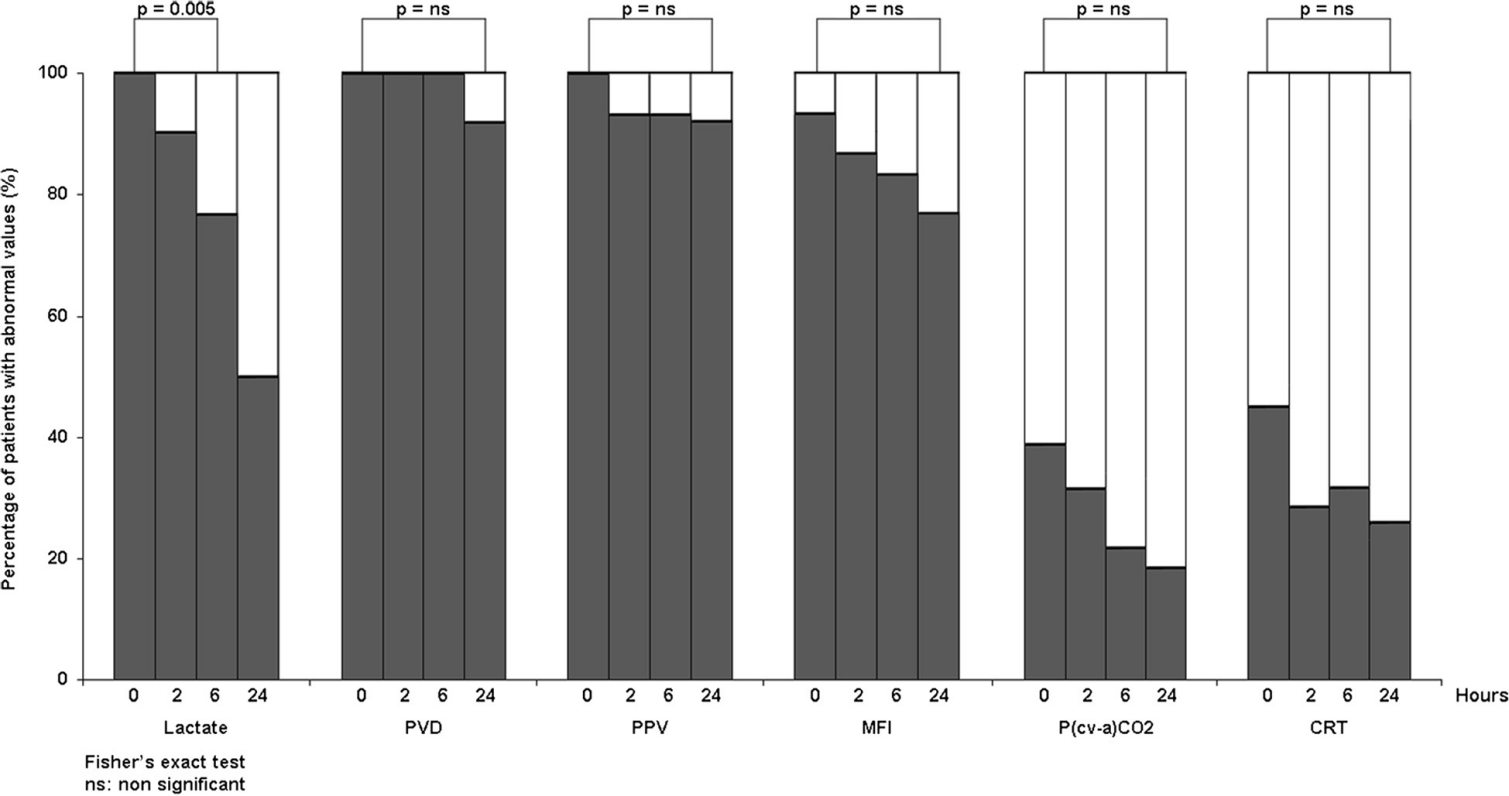
**Percentage of abnormal values for several perfusion and microcirculatory parameters in septic shock survivors.** The evolution of these parameters at different time-points during the first 24 h of intensive care unit-based resuscitation is presented. PVD, perfused vessel density; PPV, proportion of perfused vessels; MFI, microvascular flow index; P(cv-a)CO_2_, central venous-arterial pCO_2_ gradient; CRT, capillary refill time.

Medians values for microcirculatory variables and the percentage of normalization for lactate, P(cv-a)CO_2_, CRT, and microcirculatory variables at different time-points for the subgroup of patients arriving within the first 2 h of septic shock are shown in Table 3 and Figure 2, respectively. In these patients, lactate levels dropped to normal in 52% of the patients at 24 h. During follow-up of the 40 remaining patients with persistent hyperlactatemia at 24 h, 24 normalized lactate at 48 h, ten at 72 h, and six up to the seventh day. In contrast, microcirculatory variables remained abnormal in the majority of the patients, even at 24 h (Figure 2). No difference in SOFA scores (9 [7 to 11] vs. 11 [8 to 15]; *p* = 0.2) or NE requirements (0.01 [0 to 0.04] vs. 0.17 [0 to 0.24]; *p* = 0.16) was observed between patients that had normalized lactate or not at 24 h.

**Table 3:** Evolution of microcirculatory parameters in a cohort of 31 hospital survivors

**Perfusion parameters**	**Number of patients with altered baseline values**	**Baseline**	**2 h**	**6 h**	**24 h**	***p*** **value**^**a**^
PPV (%)	30	69 [62 to 75]	70 [68 to 78]	71 [67 to 79]	77 [68 to 83]	0.04
MFI (score)	28	1.9 [1.5 to 2.2]	2.0 [1.6 to 2.2]	2.1 [1.8 to 2.3]	2.2 [2.0 to 2.5]	0.003
StO_2_ (%)	8	72 [65 to 74]	73.5 [71 to 76]	75.5 [69 to 84]	77 [68 to 85]	NS
StO_2_ recovery slope (%/s)	23	1.72 [0.6 to 2.0]	1.76 [0.7 to 2.7]	1.70 [1.2 to 2.8]	2.0 [1.6 to 3.1]	0.055

Values are expressed as median [interquartile range]. ^a^Comparison within group of variables was made with non-parametric trend. PPV, proportion of perfused vessels; MFI, microcirculatory flow index; PVD, perfused vessel density; StO_2_, tissue oxygen saturation.

## 4 Discussion

Perfusion-related variables exhibit markedly different normalization rates in septic shock survivors, most of them exhibiting a biphasic response with an initial rapid improvement, followed by a much slower trend thereafter. This fact should be taken into account to determine the most appropriate criteria to stop resuscitation opportunely and avoid the risk of over-resuscitation.

Central venous oxygen saturation, P(cv-a) CO_2_, and CRT values were already normal in the majority of patients at ICU admission after some previous volume loading, and it appears that these variables are particularly responsive to DO_2_ increasing maneuvers. In a previous study, ScvO_2_, increased from 49% to 77% in septic shock patients subjected to early aggressive DO_2_ optimization [3]. The sensitivity of ScvO_2_ to pre-ICU fluid loading probably explains the almost negligible incidence of low ScvO_2_ values in the ICU setting [25],[26]. CRT may also improve rapidly after fluid resuscitation, and we found that normality of CRT values increased from 46% to 70% after 2 h of resuscitation. Changes in ScvO_2_, P(cv-a) CO_2_, and CRT appeared to become slower after 6 h, eventually representing the influence of non-flow dependent mechanisms on the remaining abnormalities [6],[27],[28].

The case of hyperlactatemia is paradigmatic. Although tissue hypoperfusion has been traditionally considered the most common cause of hyperlactatemia, there is increasing evidence for concomitant non-hypoxic and thus, non-flow dependent mechanisms [28],[29] that may influence the time course of lactate recovery rate. The distinction between these two scenarios (flow-responsive vs. non-flow dependent hyperlactatemia) should strongly impact the therapeutic approach [13].

As an example, treatment of the latter with sustained efforts aimed at increasing DO_2_ could lead to detrimental effects of excessive fluids or inotropes. In our study, lactate exhibited a significant decrease of almost 50% of basal median values during the first 6 h of resuscitation associated with a rapid normalization of other metabolic and peripheral perfusion parameters (Figure 1). However, further decrease in lactate was very slow since lactate of 48% of patients was normalized beyond the first ICU day. This behavior may raise a doubt whether these patients would have benefited from more volume loading. Nevertheless, as negative dynamic predictors or a plateau Starling curve discarded further fluid responsiveness, we had no objective evidence that persistent hyperlactatemia could be addressed to ongoing flow dependent mechanisms. Thus, it appears that lactate decrease can be characterized by a biphasic evolution: an early rapid response followed by a later slower recovery trend potentially explained by non-flow dependent mechanisms. Indeed, a recently published therapeutic algorithm focused lactate-driven resuscitation exclusively in the first 8 h of ICU management with a significant favorable impact on outcome [30].

In the present study, sublingual microcirculatory variables exhibited the slowest recovery rate. Moreover, since concomitant clinical and metabolic perfusion variables were already normal in the great majority of our patients, it appears as highly unlikely that persistent microcirculatory abnormalities may respond to additional fluids or DO_2_ optimization maneuvers after 24 h of resuscitation. In fact, fluid loading after 48 h of sepsis failed to improve microcirculatory derangements in a recent report [31]. The time-course of microcirculatory recovery during septic shock resuscitation may also follow a biphasic pattern with an early apparently flow-responsive phase [32],[33]. However, further improvements appear to be much slower with full recovery taking several days [8],[34]. The recovery slope of StO_2_ after a VOT maneuver was moderately abnormal in all patients, and like microcirculatory derangements, it showed a very slow recovery trend without significant improvement at 24 h. Taken together, our current and previous data suggest that persistent microcirculatory abnormalities after 24 h of resuscitation may represent different pathogenic mechanisms not responsive to DO_2_ increasing maneuvers.

How can we conceptualize our results? The critical decision to stop resuscitation is complex and should probably be taken after a multimodal perfusion assessment has been performed. The normalization of some variables such as ScvO_2_, lactate, or CRT is clearly a good signal, but eventually their normalization trend is more important than absolute values at certain periods. In practical terms, resuscitation would have been stopped at admission in 90% of these patients by using the single criterion of a normal ScvO_2_ but only in 52% of patients at 24 h by using a normal lactate criterion. Our study was not designed to establish which criterion is better, but it does suggest that the length of septic shock resuscitation may vary dramatically depending on the selected perfusion goal and that the different potential targets are not equivalent or interchangeable as suggested by a recent guideline [4]. Since ours is only a hypothesis-generating study, these findings should be explored and confirmed in future studies, since as mentioned before an excess in resuscitation efforts may lead to severe side effects.

Finally, due to the complexity of the pathogenic mechanisms influencing each of the perfusion variables, and of their dynamic characteristics, it is clear that treatment of septic shock may also be guided by the presence of comorbidities, adequacy of source control, and the degree of systemic inflammation, among others.

We acknowledge several potential limitations of our study. This cohort exhibited a low mortality rate. Therefore, our findings may not be universally extrapolated. However, several recent studies report a mortality of around 20% in septic shock patients undergoing early resuscitation, especially when the main source is abdominal [35]–[37]. Second, the study period may be considered not long enough and the selected time points are arbitrary. Third, this is a single center study that limits its application to other settings and centers. Fourth, due to the design of this study, we cannot determine if changes in perfusion variables over time can be ascribed to the natural evolution of disease or to the effects of some specific treatment. Fifth, although our findings suggest that the length of the resuscitation process could vary dramatically according to the selected perfusion goal, we do not know if this effectively leads to over-resuscitation in some cases since our study was not designed to establish this point. Finally, since only surviving patients compose our cohort, we know that the main endpoint of any septic shock resuscitation strategy was successfully achieved. However, we do not know if additional resuscitation could have further reduced morbidity. Nevertheless, the lack of difference in 24-h SOFA scores or NE requirements between patients who normalized lactate vs. those who did not makes this possibility unlikely. Furthermore, as dynamic predictors of fluid responsiveness were persistently negative, there is no evidence that additional resuscitation could have benefited secondary outcomes, especially considering controversial data concerning other potential therapies such as dobutamine or nitroglycerine [38],[39].

## 5 Conclusions

In conclusion, these results demonstrate that perfusion-related variables exhibit markedly different normalization rates in septic shock survivors, most of them showing a biphasic response with an initial rapid improvement, followed by a much slower trend thereafter. The length of septic shock resuscitation may vary dramatically depending on the selected perfusion goal. This fact should be taken into account to determine the most appropriate criteria to stop resuscitation opportunely and avoid the risk of over-resuscitation.

## Abbreviations

APACHE: Acute physiology and chronic health evaluation

CVP: central venous pressure

CRT: capillary refill time

DO_2_: oxygen transport

IAP: intra-abdominal pressure

ICU: intensive care unit

MAP: mean arterial pressure

MFI: microcirculatory flow index

NE: norepinephrine

NIRS: near-infrared spectroscopy

P(v-a)CO_2_: mixed venous to arterial pCO_2_ gradient

PPV: proportion of perfused vessels

PVD: perfused vascular density

SOFA: Sequential Organ Failure Assessment

ScvO_2_: central venous oxygen saturation

StO_2_: tissue oxygen saturation

SvO_2_: mixed venous oxygen saturation

VOT: vascular occlusion test

## Competing interests

The authors declare that they have no competing interests.

## Authors’ contributions

GH, CL, and AB conceived the study, participated in its design and coordination, and helped to draft the manuscript. JB, CI, GF, and GO conceived the study and helped to draft the manuscript. RC helped to draft the manuscript and performed statistical analyses. EK maintained the database, performed statistical analyses, and designed tables and figures. AF registered and analyzed microcirculatory images. CR recruited and followed up patients. TR recruited and followed up patients. All authors read and approved the final manuscript.

## Additional file

## Supplementary Material

Additional file 1: Table S1. Hemodynamic and perfusion-related parameters in 20 non-survivors. Click here for file
