# Supplementary material for: When to stop septic shock resuscitation: clues from a dynamic perfusion monitoring
Source: Ann Intensive Care. 2014 Oct 11;4:30. doi: 10.1186/s13613-014-0030-z (PMC4273696; doi:10.1186/s13613-014-0030-z)
Supplement: Additional file 1: Table S1. — Hemodynamic and perfusion-related parameters in 20 non-survivors. [file s13613-014-0030-z-S1.docx]

**Additional file 1: Table S1 Hemodynamic and perfusion-related parameters in 20 non-survivors**

| Parameter | 0 h | 2 h | 6 h | 24 h |
| --- | --- | --- | --- | --- |
| Lactate (mmol/L) | 3.0 [2.4-15.1] | 2.9 [1.8-14.9] | 3.1 [1.8-13.1] | 2.7 [1.9-12.3] |
| NE dose (mcg/kg/min) | 0.19 [0.07-0.52] | 0.18 [0.07 -0.52] | 0.25 [0.04 – 0.71] | 0.45 [0.06 – 0.82] |
| P(cv-a)CO_2_ (mmHg) | 4 [2-6] | 3 [1-4] | 4 [1-20] | 5 [0-11] |
| ScvO_2_ (%) | 77 [75-88] | 82 [79-90] | 82 [78-91] | 81 [75-91] |
| CRT (s) | 4 [2-6] | 3 [2-6] | 4 [2-5] | 2 [2-2] |

Values expressed as Median [interquartile range].

NE, norepinephrine; p(cv-a)CO_2_, central venous to arterial pCO_2_ gradient, ScvO_2_, central venous oxygen saturation; CRT, capillary refill time.
